# Spontaneous Selective Preconcentration Leveraged by Ion Exchange and Imbibition through Nanoporous Medium

**DOI:** 10.1038/s41598-018-38162-6

**Published:** 2019-02-20

**Authors:** Dokeun Lee, Jung A. Lee, Hyomin Lee, Sung Jae Kim

**Affiliations:** 1 Department of Electrical and Computer Engineering, Seoul National University, Seoul, 08826 Republic of Korea; 2 Department of Chemical & Biological Engineering, Jeju National University, Jeju, 63243 Republic of Korea; 3 Inter-university Semiconductor Research Center, Seoul National University, Seoul, 08826 Republic of Korea; 4 Nano Systems Institute, Seoul National University, Seoul, 08826 Republic of Korea

## Abstract

Manipulating mechanism of particle’s motion has been extensively studied for the sample preparation in microfluidic applications including diagnostics, food industries, biological analyses and environmental monitoring. However, most of conventional methods need additional external forces such as electric field or pressure and complicated channel designs, which demand highly complex fabrication processes and operation strategies. In addition, these methods have inherent limitations of dilution or mixing during separation or preconcentration step, respectively, so that a number of studies have reported an efficient selective preconcentration process, *i*.*e*. conducting the separation and preconcentration simultaneously. In this work, a power-free spontaneous selective preconcentration method was suggested based on leveraging convective flow over diffusiophoresis near the water-absorbing nanoporous ion exchange medium, which was verified both by simulation and experiment. Especially, the velocity of the convective flow by an imbibition deviated from the original tendency of *t*^−1/2^ due to non-uniformly patterned nanoporous medium that has multiple cross-sectional areas. As a result, the direction of particle’s motion was controlled at one’s discretion, which led to the spontaneous selective preconcentration of particles having different diffusiophoretic constant. Also, design rule for maximizing the efficiency was recommended. Thus, this selective preconcentration method would play as a key mechanism for power-free lab on a chip applications.

## Introduction

Separation and preconcentration are the key sample preparation step in a wide range of chemical, environmental, and biological processes. Especially, those sample preparation technologies using distinct physical properties of particles or cells are particularly important in microfluidic applications including diagnostics^1–4^, food industries^5–7^, biological analyses^8,9^ and environmental monitoring^10,11^. However, these processes have mutual contradiction since aggressive separations inevitably dilute a sample and sample preconcentration mixes all components in the sample so that a number of studies aim to develop an efficient selective preconcentration process, *i*.*e*. conducting the separation and preconcentration simultaneously^12–14^. Previously developed selective preconcentration methods usually require external stimuli such as electric field^14–16^, magnetic field^4,17,18^, acoustic waves^19–21^, *etc*. However, these active selective preconcentration methods highly demand complex/expensive fabrication processes and chip design for the external forces, preventing the practical applications where energy input is of critical concern. This has resulted in the development of several passive method without any external forces, for example, capillary force^22,23^, pinched flow fractionation^24–26^, inertial focusing^4,27,28^, and inertia-elastic focusing^29–31^, *etc*. However, complicated microchannel design or specific chemicals are often required for their processes, which induces practical difficulty of real applications. In addition, external pump is still utilized in order for careful control of the input flow rate. It means that these methods are unsuitable to portable and cost-effective devices.

Recently, diffusiophoresis have been suggested as the alternative method of manipulation of particles without external forces^32–38^. Diffusiophoresis refers to the manipulation of particle’s motion with a concentration gradient induced either by two miscible liquids of different concentrations^39–43^ or by ion exchange^44–48^. Similar with our mechanism, two studies have reported about utilizing the balance between diffusiophoresis and convective flow for separating/preconcentrating colloid particles. Using the concentration gradient generated by injecting two different concentrations of electrolyte, Friedrich *et al*. and Ault *et al*. reported that colloidal particles could be preconcentrated and multiple particles could be separated, respectively^36,49^. However, these works should still need additional apparatus for the concentration gradient generation and external pressure-driven pump to induce convective flow.

Meanwhile, the concentration gradient induced by ion exchange is spontaneously generated if the ion exchange medium instantly meets the solution containing non-protonic cation. When the nanoporous medium is wetted with water, surface group on the medium is dissociated. The proton inside the medium would be exchanged with non-protonic cation dissolved in water through the Brownian motion^50^. It was reported that the ion concentration near the medium was unchanged because ion exchange process is 1:1 process^51^. However, due to the difference of diffusivity between proton and cation, natural ion depletion layer is generated near the nanoporous medium^45,52,53^. In the presence of concentration gradient near the medium, the electrical double layer of a charged particle is deformed and internal electric field is induced around the particle. Thus, the particle is effectively transported by the diffusiophoresis.

**Figure 1:**
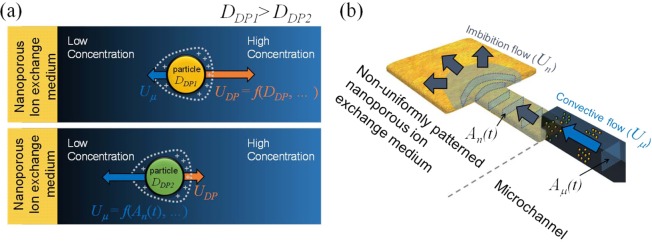
(**a**) Schematics of concentration gradient generated near the nanoporous ion exchange medium. The direction of negatively charged particle’s motion was affected by *U*_*DP*_ and *U*_*μ*_. When *U*_*DP*_ > *U*_*μ*_, particle moves away from the medium (top), and *vice versa* (bottom). (**b**) Schematic of non-uniformly patterned nanoporous ion exchange medium, and the imbibition through the increasing *A*_*n*_ as a function of time.

However, water permeance through the nanoporous medium in such diffusion-dominant environment should be considered since the medium should absorb the water to have the dissolved protons which will be exchanged with other cations^53^. Considering the water permeance through the nanoporous medium, the concentration profile near the medium has three types (ion depletion, ion accumulation and intermediate) depending on the permselectivity and the water-permeability of the medium^53^. Under the medium that has sufficiently low water permeance, a charged particle’s moving velocity (*U*_*p*_) is independently affected by two velocities as shown in Fig. 1(a): diffusiophoretic velocity (*U*_*DP*_) and convective velocity (*U*_*μ*_). Thus *U*_*p*_ is expressed by the sum of two flow velocities as

1 $${U}_{p}={U}_{DP}+{U}_{\mu }$$

where

2 $${U}_{DP}={D}_{DP}\nabla \,\mathrm{log}\,{c}_{tot}$$

and

3 $${{U}}_{\mu }={\phi }_{p}\frac{{An}}{{{A}}_{\mu }}{{U}}_{n}={\phi }_{p}\frac{{{A}}_{n}}{{{A}}_{\mu }}\sqrt{\frac{{S}}{{t}}}$$

Here, diffusiophoretic constant, *D*_*DP*_ is already a function of the zeta potential of a particle, viscosity of fluid, temperature, diffusivity of ions^45,53,54^ and the radius of a particle^43^, and *c*_*tot*_ is the total ion concentration. It is reported that *U*_*DP*_ is proportional to *t*^−1/2^ ^48^. *A*_*n*_ is the cross-sectional area of a wetted nanoporous medium, *A*_*μ*_ is the cross-sectional area of a micro-channel, *φ*_*p*_ is the porosity of a nanoporous medium, *S* is the absorbing parameter^53^, *U*_*n*_ is the velocity of wetting through nanoporous medium obtained by Darcy’s law, and *t* is the time. *U*_*μ*_ is derived from the flow continuity condition at the interface between the nanoporous medium and the micro-channel. Both of the velocities in Eq (2–3) are inversely proportional to the square root of *t*, so that the direction of the particle’s motion should be unidirectional. However, *U*_*μ*_ is able to be manipulated when nanoporous medium is non-uniformly patterned as shown in Fig. 1(b). Owing to the non-uniformity, *A*_*n*_ is no longer constant and becomes a function of *t* since the water is absorbed through the expanding pathway as shown in Fig. 1(b). Consequently, *U*_*μ*_ becomes a saturating function other than *t*^−1/2^ ^55–59^, so that the direction of the particle’s motion is able to be leveraged. In this work, by adjusting the *U*_*μ*_, manipulating motion of multiple particles was presented and the spontaneous selective preconcentration mechanisms were verified both by simulation and experiment. Moreover, our method used simple straight and dead-end microchannel design and require no externally connected devices such as pump or electric power source so that it would be an effective mean to be developed as a portable analytical device in resource-limited settings.

## Results and Discussions

### Mechanism of leveraging convective flow over diffusiophoresis by non-uniformly patterned nanoporous medium

**Figure 2:**
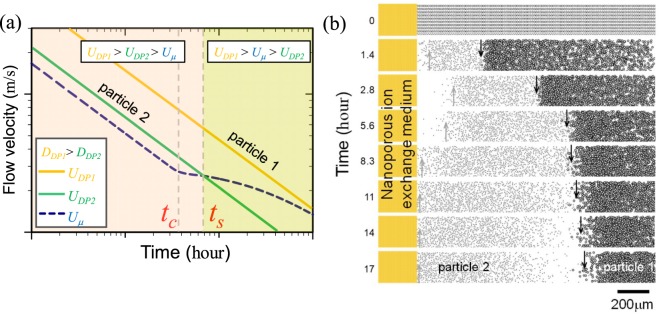
(**a**) The plot of *U*_*DP*_s of particle 1 and 2 having different diffusiophoretic constant (*U*_*DP*1_ > *U*_*DP2*_) and *U*_*μ*_ induced by non-uniformly patterned nanoporous medium along the time axis. (**b**) The Langevin dynamics simulation of particle movement under the concentration gradient and convective flow field. See Supplementary Video 1.

With the non-uniformly patterned nanoporous ion exchange medium as depicted in Fig. 1(b), the flow velocity induced by the imbibition through the medium (*U*_*μ*_) is numerically estimated in Fig. 2(a). The simulated flow velocity is calculated based on the Richard’s equation^59^ and more information on the simulation is available in Supplementary Note 1. Until the critical time (*t*_*c*_), the flow velocity follows the 1-dimensional Darcy’s law since the water is absorbed through the fixed *A*_*n*_. After *t*_*c*_, however, the flow velocity deviates from the straight line and leads to the saturated velocity due to the increasing *A*_*n*_ with an expanding water-pathway^57^. In Fig. 2(a), diffusiophoretic constant of particle 1 was higher than that of particle 2, and the comparison among the velocities was *U*_*DP*1_ > *U*_*DP2*_ > *U*_*μ*_, before the time of direction switching (*t*_*s*_), which meant that both particles would move from the nanoporous medium to the bulk. After *t*_*s*_, the comparison among the velocities was *U*_*DP*1_ > *U*_*μ*_ > *U*_*DP2*_, which meant that particle 1 would still move to the bulk but the particle 2 would reversely move to the nanoporous medium. Thus, the particles would be separated due to their different moving direction without external power.

Langevin dynamics (LD) simulation was conducted for confirming this separation. The force balance for each particle included the Brownian motion of a particle itself and the drag force from *U*_*μ*_ and *U*_*DP*_. Figure 2(b) showed the LD results with the black particles representing the particle 1 and the gray particles representing the particle 2. The critical time (*t*_*c*_) when saturated flow velocity started were set to be 10,000 seconds (2.8 hours). Before *t*_*c*_, both particles were pushed away from the ion exchange medium due to the diffusiophoretic force. During this period, particles near the ion exchange medium were depleted since the diffusiophoretic force were stronger near the medium. At the same time, particles near the reservoir were drawn towards the medium by the fluid flow. Due to these two forces acting simultaneously, particles were not only depleted from the medium but also preconcentrated at the depletion boundary marked as arrows in Fig. 2(b). These depletion boundaries extended proportional to *t*^1/2^ until *t*_*c*_ and the particle 1 having the higher diffusiophoretic constant were depleted further than particle 2. Between *t*_*c*_ and *t*_*s*_, the depletion boundaries of both types of particle gradually stopped developing because of the saturated *U*_*μ*_. After *t*_*s*_ (which was estimated to be around 18,000 seconds (5 hours) in the simulation), only particle 2 (gray) which had the lower diffusiophoretic constant switched their direction of motion towards the medium, while particle 1 (black) kept its direction toward reservoir, leading to a simultaneous separation and preconcentration, *i*.*e*. selective preconcentration. Details on the simulation are available in Supplementary Note 2 and see Supplementary Video 1.

### Experimental demonstration of particles’ selective preconcentration

**Figure 3:**
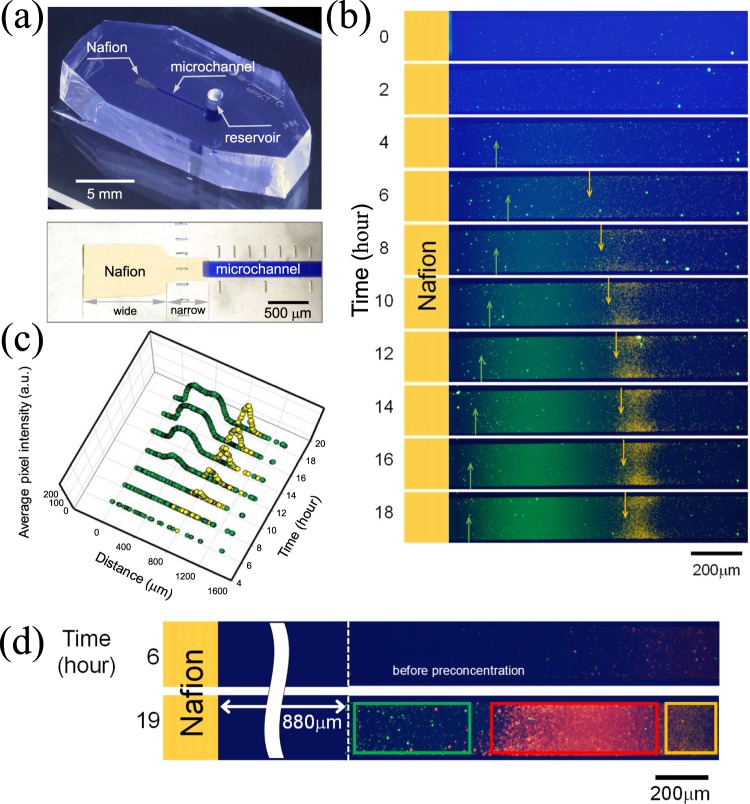
(**a**) The image of an assembled microfluidic chip and the microscopic view of the chip. (**b**) Time-revolving images of two types of particles’ selective preconcentration. See Supplementary Video 2. (**c**) Average pixel intensity of separated yellow particles and green particles as a function of distance and time. (**d**) The experimental demonstration of three types of particles’ selective preconcentration. See Supplementary Video 3.

Aforementioned LD analysis was experimentally demonstrated as shown in Fig. 3. Nafion as a nanoporous medium was used because Nafion has high perm-selectivity with the low water permeance for the generation of ion depletion near the medium^53^. The microfluidic device made of polydimethyl-siloxane (PDMS) had a microchannel that was connected with non-uniformly patterned Nafion at the end of the channel and a reservoir at the other end (Fig. 3(a)). The microchannel was filled with 1 mM KCl electrolyte solution containing the carboxylate functionalized fluorescent particles (negatively charged and the diameters of 2 μm and 0.04 μm). Larger particle has higher diffusiophoretic constants^60^. In Fig. 3(b), the yellow particles (2 μm) with relatively high diffusiophoretic constant gradually moved to the bulk. However, the green particles (0.04 μm) with relatively low diffusiophoretic constant moved to the bulk until around 6 hr but after that time, they moved back towards the ion exchange medium as arrows indicated. Moreover, in the region of constant concentration (*i*.*e*. near reservoir), particles were forced only by convective flow so that particles were continuously provided from the reservoir, leading an efficient preconcentration. In Fig. 3(c), the intensity of each particle was plotted by analyzing the fluorescent signal. As time passed, the distance between the peaks of yellow particles and green particles increased (separation), and the intensity of each particle simultaneously increased (preconcentration). See Supplementary Video 2. The separation resolution (*R*_*s*_) calculated by (peak to peak distance)/(average width of bands) led to 1.21^61^. This number indicates almost perfect separation (*i*.*e*. no overlap) under assumption that the distribution follows Gaussian distribution. In addition, the average pixel intensity of yellow particle reached up to 171.24 at 18 hr from 0.50 at 6 hr, which denotes that yellow particles were preconcentrated over 300-fold. Also, the average pixel intensity of green particle reached up to 171.28 at 18 hr from 1.83 at 6 hr, which denotes that yellow particles were preconcentrated over 90-fold. In this sense, our method is comparable to previous researches utilizing an external electric field to selective-preconcentrate multiple particles^14,15,62^ if only preconcentration factor was considered. This comparison was irrelevant if a short operation time is demanded.

Selective preconcentration of three types of particles was also demonstrated in Fig. 3(d) using the red particle of 0.2 μm which had intermediate diffusiophoretic constant between yellow particle and green particle (*i*.*e*. *D*_*DP*_yellow_ > *D*_*DP*_red_ > *D*_*DP*_green_). As expected, three types of particles were selectively preconcentrated in the order of diffusiophoretic constant value. See Supplementary Video 3.

### Design rule for maximizing efficiency of selective preconcentration

As mentioned earlier, when the nanoporous medium was being wetted through the straight portion of nanoporous medium, the *U*_*μ*_ followed 1-dimensional Darcy’s law due to the constant *A*_*n*_ fixed as *A*_*n*,*0*_. Then, *A*_*n*_ increased due to the expanding water-pathway would cause *U*_*μ*_ to be saturated. That is, *U*_*μ*_ was described as

4 $${U}_{\mu }={\varphi }_{p}\frac{{{\rm{A}}}_{{\rm{n}}{,}0}}{{A}_{{\mu }}}\sqrt{\frac{{S}}{{t}}}\,{\rm{when}}\,0 < t < {t}_{c}$$

and

5 $${U}_{\mu }={\varphi }_{p}\frac{{{\rm{A}}}_{{\rm{n}}{,}0}}{{A}_{{\mu }}}\sqrt{\frac{{S}}{{{t}}_{{c}}}}\,{\rm{when}}\,{t}_{c} < t < {t}_{s}.$$

Due to the saturated *U*_*μ*_, it intersected with the *U*_*DP*_ at the time of direction switching (*t*_*s*_) as

6 $${{t}}_{{s}}=\frac{{t}_{c}}{S}{(\frac{{D}_{DP}}{{c}_{tot}}\frac{\partial {c}_{tot}}{\partial \eta }\frac{{{A}}_{\mu }}{{\varphi }_{{p}}{{A}}_{n}})}^{{2}}.$$

**Figure 4:**
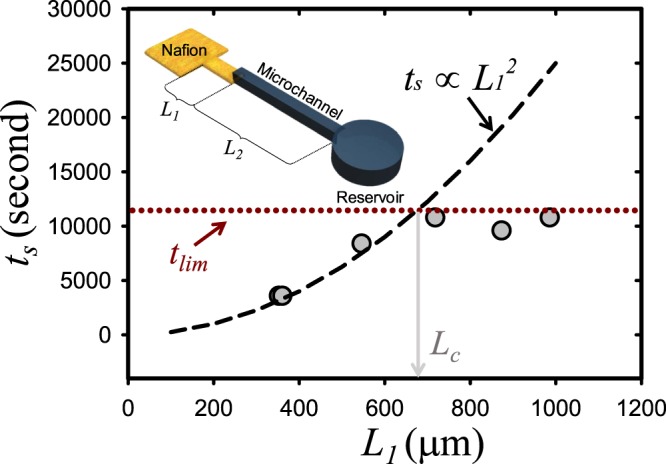
The quantitative analysis on the relationship between *t*_*s*_ and *L*_*1*_.

Here, *t*_*c*_ is a function of *L*_1_ (the length of the straight region prior to the flow expanding pathway as shown in the inset of Fig. 4) according to the Darcy’s law as

7 $${t}_{c}=\frac{{{L}_{1}}^{2}}{4S}.$$

Thus, the relationship between *t*_*s*_ and *L*_1_ was obtained as

8 $${{t}}_{s}={(\frac{{D}_{DP}}{{c}_{tot}}\frac{\partial {c}_{tot}}{\partial \eta }\frac{{{A}}_{\mu }}{{\phi }_{{p}}{{A}}_{n}}\frac{1}{2S})}^{{2}}{{L}_{1}}^{2},$$

and indicated by a dashed line as shown in Fig. 4, which was experimentally verified by adjusting *L*_1_ (see gray circles). Using the microchip, *t*_*s*_ was obtained by measuring the time when the particles (carboxylate 0.2 μm) switched their moving direction. The experimental values of *t*_*s*_ were proportional to *L*_1_^2^ until *L*_*1*_ = 700 μm, but were saturated at a certain constant for *L*_*1*_ longer than the critical length (*L*_*c*_) due to the diffusion of ions from the reservoir. The diffusion from the reservoir mitigated the concentration gradient formed by the ion exchange, which was usually neglected since the length of microchannel (*L*_2_) was commonly assumed as infinitely long. Considering the diffusion from the bulk, the restoration of total concentration was simulated in Supplementary Note 3. The experimentally estimated time of limitation (*t*_*lim*_) when the particles changed their moving direction without the help of increasing *A*_*n*_ was around 14,000 seconds. That is, *t*_*s*_ would be limited to *t*_*lim*_ when *L*_1_ was longer than *L*_*c*_, which meant that the effect of *L*_2_ became dominant over *L*_1_. Conclusively, *L*_1_ is the value for determining the time of starting separation and, thus, it should be chosen smaller than *L*_*c*_, for maximizing the efficiency of selective preconcentration of particles.

## Conclusions

Selective preconcentration plays an important role for sample preparation step in a wide range of biochemical microfluidic applications. However, conventional selective preconcentration methods usually require additional devices (or apparatus) for inducing external stimuli such as electric field or pressure. This has resulted in the necessity and development of power-free selective preconcentration mechanism. In this work, spontaneous selective preconcentration method was presented based on leveraging convective flow induced by imbibition through nanoporous medium over diffusiophoresis. While traditional imbibition vs. diffusiophoresis mechanism conveyed a unidirectional force field, we successfully demonstrated a bidirectional field utilizing non-uniformly patterned nanoporous medium. These mechanisms were verified both by simulation and experiment. Consequently, the selective preconcentration of two or three particles having different diffusiophoretic constant was demonstrated and a design rule was also suggested through a simple analysis for maximizing the efficiency of power-free selective preconcentration. Although these mechanisms have the limitation of slow processing in comparison to other methods using external fields, this method can be useful for a time-insensitive lab on a chip application such as environmental monitoring and food monitoring, *etc*. In order to overcome this drawback, one could employ a paper device that has faster imbibition. In addition to this, our method is unsuitable for recovering the selective preconcentrated sample due to the dead-end channel. However, we are expecting the applications of this work for the portable diagnosis devices where one can directly observe the selectively preconcentrated sample inside the channel.

## Materials and Methods

As building blocks of each microchannel for filling samples and Nafion (Sigma Aldrich, USA), polydimethyl-siloxane (PDMS, Sylgard 184 silicone elastomer kit, Dow Corning, USA) was used as shown in Fig. 3(a). PDMS base and curing agent (Sylgard 184 silicone elastomer kit, Dow Corning, USA) were mixed in a ratio of 10:1 and degassed in a vacuum chamber for one hour. The mixed solution was poured onto a silicon wafer which had patterned microchannels and was cured in an oven for four hours at 75 °C.

The Nafion as a nanoporous medium was patterned on the glass slide following a previously reported surface patterning method with the prepared PDMS block for filling Nafion^63,64^. The Nafion is divided into two regions of a narrow Nafion (dimension: width 200 μm × depth 0.5 μm × length 500 μm) and a wide Nafion (dimension: width 600 μm × depth 0.5 μm × length 1,100 μm). On the Nafion-patterned glass slide, the prepared PDMS block for filling samples was irreversibly bonded to a designated position using plasma bonder (CuteMP, Femto Science, Korea) as shown in Fig. 3(a). The microchannel has the dimension of width 200 μm × depth 15 μm × length 5 mm and filled with a mixture of 1 mM KCl solution (Sigma Aldrich, USA) with the negatively charged fluorescent carboxylate particles (diameter = 2 μm, 0.2 μm, and 0.04 μm, Invitrogen, USA). The motions of fluorescent particles were captured by an inverted fluorescence microscope (IX53, Olympus, Japan) and the CellSens program (Olympus, Japan). The fluorescent intensity was analyzed by ImageJ.

## Supplementary information

SI_Video1_Langevin dynamics simulation

SI_Video2_selective preconcentration_two particles

SI_Video3_selective preconcentration_three particles

Supporting information
