# Supplementary material for: Spontaneous Selective Preconcentration Leveraged by Ion Exchange and Imbibition through Nanoporous Medium
Source: Sci Rep. 2019 Feb 20;9:2336. doi: 10.1038/s41598-018-38162-6 (PMC6382859; doi:10.1038/s41598-018-38162-6)
Supplement: Supplementary file 4 — Supporting information [file 41598_2018_38162_MOESM4_ESM.pdf]

Supplementary Note for

Spontaneous Selective Preconcentration Leveraged by Ion Exchange and Imbibition through Nanoporous Medium

Dokeun Lee, Jung A Lee, Hyomin Lee and Sung Jae Kim

Supplementary Note 1. Numerical analysis of the 2-dimensional imbibition through a nanoporous medium

Imbibition of liquid in a two-dimensional porous medium is described by the Richard's equation as [1]

$$\frac{\partial \theta}{\partial t} = \nabla \cdot \left(D\frac{\partial \theta}{\partial x}\mathbf{e}_x + D\frac{\partial \theta}{\partial y}\mathbf{e}_y \right) = D\nabla^2\theta, \tag{S1}$$

where θ is the volumetric content of liquid defined as the volume of liquid divided by the void volume of the porous medium, t is the time, x and y are the spatial coordinates, and D is the equivalent diffusivity. Here, D is defined as

$$D \equiv \frac{k}{\mu}\frac{\partial P}{\partial \theta}, \tag{S2}$$

where P is pressure, k is the permeability, μ is the viscosity of the liquid. This equivalent diffusivity connects the Richard's equation with the conventional Darcy's law which explains the imbibition of liquid using the pressure gradient generated from the capillary pressure at the imbibition head. However, unlike the convectional Darcy's law, the permeability (k) and capillary pressure (P_c) are not constant but highly dependent on θ. Therefore, the equivalent diffusivity (D) is a function of θ as

$$D = D_0\theta^n, \tag{S3}$$

where the exponent n is related to the pore size distribution.

Using the Eq. (S1), the imbibition through a nonuniformly patterned nanoporous medium with a varying cross-sectional area was numerically simulated using COMSOL commercial software (COMSOL 4.3a) and the flow velocity was estimated by averaging the influx of water through the bottom wall. The water absorption through the patterned medium was visualized in Fig. S1, with the simulation conditions of L_1 = 10^{-3} m, D_0 = 10^{-9} m^2/s, n = 6, and the characteristic time scale was estimated as $\tau_c = L_c^2/D_0$.

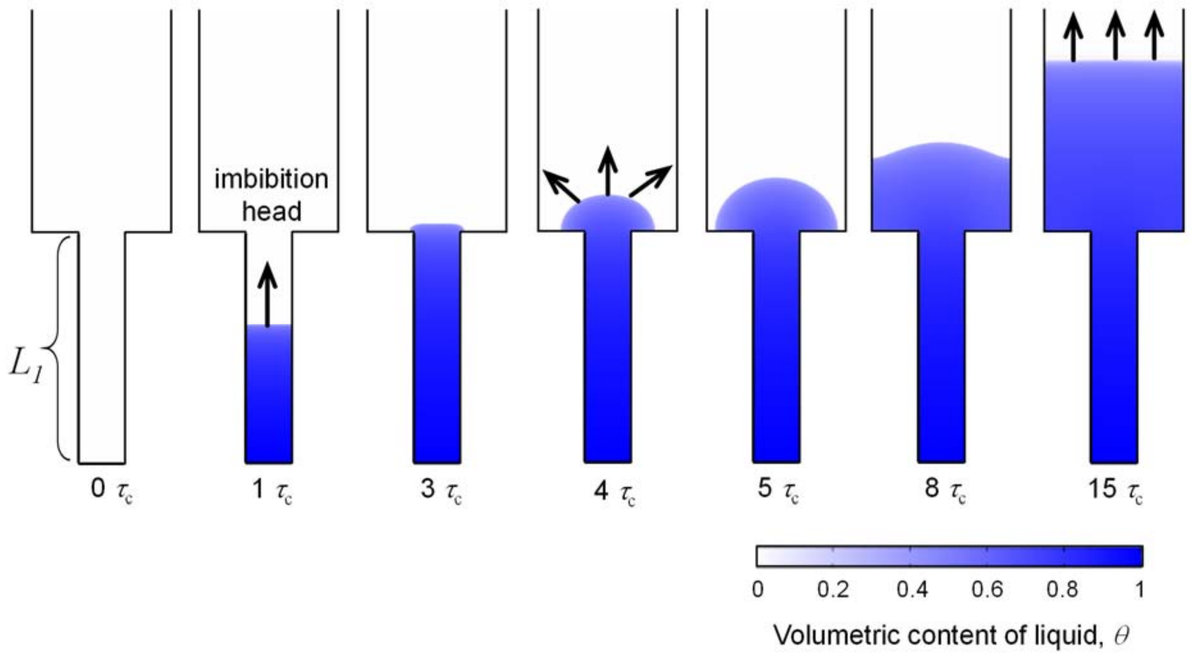

Figure S1. Numerical simulation results of imbibition through two-dimensional porous medium.

When the water was absorbed through L_1, (*i.e.* straight part of ion exchange medium) the influx of water was proportional to $t^{-1/2}$ following the one-dimensional Darcy's law. Therefore, the slope of log-scaled graph in Fig. 2(a) in the main text was -1/2. After the imbibition head reaches the end of the L_1, the influx of the water stopped decreasing for a while due to the

widening imbibition head. Therefore, the saturated flow velocity was maintained until the imbibition head stopped widening.

Supplementary Note 2. Langevin dynamics simulation of the particle separation.

The separation of charged particles having different zeta-potential (ζ_{p1}, ζ_{p2}) and radius (a_1, a_2) was simulated using the Langevin dynamics simulation. Each particle was assumed to experience three major forces in the simulation.

The first one was the Brownian force ($\mathbf{F}_B$) which should satisfy

$$\langle\langle \mathbf{F}_B(t) \rangle\rangle = \mathbf{0} \tag{S4}$$

and

$$\langle\langle \mathbf{F}_B(t_1) \cdot \mathbf{F}_B(t_2) \rangle\rangle = 6k_B T C_f \delta(t_1 - t_2) \tag{S5}$$

from the fluctuation-dissipation theorem. Here << · >> is the ensemble average operator, t_1 and t_2 are the arbitrary time, k_B is the Boltzmann constant, T is the absolute temperature, C_f is the friction coefficient, and $\delta(t)$ is the Dirac delta function. The values of C_f were chosen for spherical particles having finite diameters.

The second one was the diffusiophoretic force which was originated from both the deformation of electrical double layer around a charged particle due to the concentration gradient and the ion diffusivity difference. Therefore, under the concentration gradient, this diffusiophoretic velocity ($\mathbf{U}_{DP}$) can be expressed as summation of chemiphoretic velocity ($\mathbf{U}_{CP}$) and electrophoretic velocity ($\mathbf{U}_{EP}$) in the case of a multi-species electrolyte (K^+,H^+ and Cl^-) as [2,3]

$$\mathbf{U}_{DP} = \mathbf{U}_{CP} + \mathbf{U}_{EP} \tag{S6}$$

where

$$\mathbf{U}_{CP} = -\frac{2\varepsilon R^2 T^2}{\mu F^2} \ln\left[1 - \tanh^2\left(\frac{F\zeta_p}{4RT}\right)\right] \nabla \ln\left(\frac{c_{tot}}{2}\right), \tag{S7}$$

and $$\mathbf{U}_{EP} = \frac{\varepsilon\zeta_p}{\mu}\frac{RT}{F}\frac{D_K\nabla c_K - D_{Cl}\nabla c_{Cl} + D_H\nabla c_H}{D_K c_K + D_{Cl} c_{Cl} + D_H c_H},\tag{S8}$$

ε is the dielectric constant, R is the gas constant, T is the absolute temperature around a particle, F is the Faraday constant, ζ_p is the zeta potential of a particle, c_{tot} is the total ion concentration. In our system, the concentration gradient was naturally formed near the ion exchange medium due to the diffusivity difference between the exchanging ionic species (K^+, H^+), and the concentration profiles of each ionic species can be expressed as [3,4]

$$c_K(x,t) = c_0 erf\left(\frac{x}{\sqrt{4D_{K,eff}t}}\right),\tag{S9}$$

$$c_H(x,t) = \sqrt{\frac{D_{K,eff}}{D_{H,eff}}}c_0\left[1 - erf\left(\frac{x}{\sqrt{4D_{H,eff}t}}\right)\right] \text{ and}\tag{S10}$$

$$c_{Cl} = c_K + c_H.\tag{S11}$$

Here, $D_{i,eff}$ is the effective diffusivity obtained from the charge neutrality condition between anions and cations and it is defined as

$$D_{i,eff} \equiv \frac{2D_i D_{Cl}}{D_i + D_{Cl}},\tag{S12}$$

where D_i, is the diffusivity of ionic species i.

The last force acting on the charged particle was the convective drag force due to the flow velocity ($\mathbf{U}_\mu$) generated from the imbibition through the nanoporous ion exchange medium, and the flow velocity was expressed as in Eq. (4-5) in main text.

Considering the Brownian motion of particle itself, the diffusiophoretic force, and the flow field, the Langevin equation under the overdamped limit can be expressed as

$$\frac{d\mathbf{r}}{dt} = \frac{\mathbf{F}_B}{C_f} + \mathbf{U}_\mu + \mathbf{U}_{DP}\tag{S13}$$

where $\mathbf{r}$ is the position vector of each particle. Interactions between particle and wall were

assumed as perfect elastic collision and the particle-particle interactions were neglected. Simulation condition of the particle 1 was $\zeta_{p1} = 6.2\ V_T$, $a_1 = 1.5\times10^{-6}$ m, and that of the particle 2 was $\zeta_{p2} = 5.6\ V_T$, $a_2 = 0.7\times10^{-6}$ m. The critical time (t_c) when the saturated $\mathbf{U}_\mu$ started was 10,000 seconds. Before t_c, the flow velocity followed the one-dimensional Darcy's law with the absorbing parameter of 3×10^{-10} m^2/s. After t_c, the saturated flow velocity lasted until it recovered to the one-dimensional Darcy's law with the absorbing parameter of 5.5×10^{-10} m^2/s.

The simulated results were shown in Fig. 2(b) in the main text, and the corresponding histograms were shown in Fig. S2. Before the critical time which was set to be 1,000 seconds, both particles near the ion exchange medium were depleted due to the diffusiophoretic force. Since the diffusiophoretic force is stronger near the ion exchange medium while it becomes negligible near the bulk reservoir, the particles near the medium moved towards the reservoir (right). At the same time, particles near the reservoir moved towards the medium (left) along the convective flow generated by the imbibition through the nanoporous ion exchange medium. This led the particles to be preconcentrated near the depletion boundary having the intensity peaks at the depletion boundaries. The depletion boundaries of particles were decided by the particles' diffusiophoretic constant, which means that the peak of the particle 1 of higher diffusiophoretic constant were formed further than that of particle 2 due to the stronger diffusiophoretic force. After t_c, the saturated flow velocity started and the depletion boundaries suddenly slowed down the extension rate. Finally at t_s which was estimated to be around 18,000 seconds from the simulation, particle 2 of lower diffusiophoretic constant changed the moving direction towards the medium.

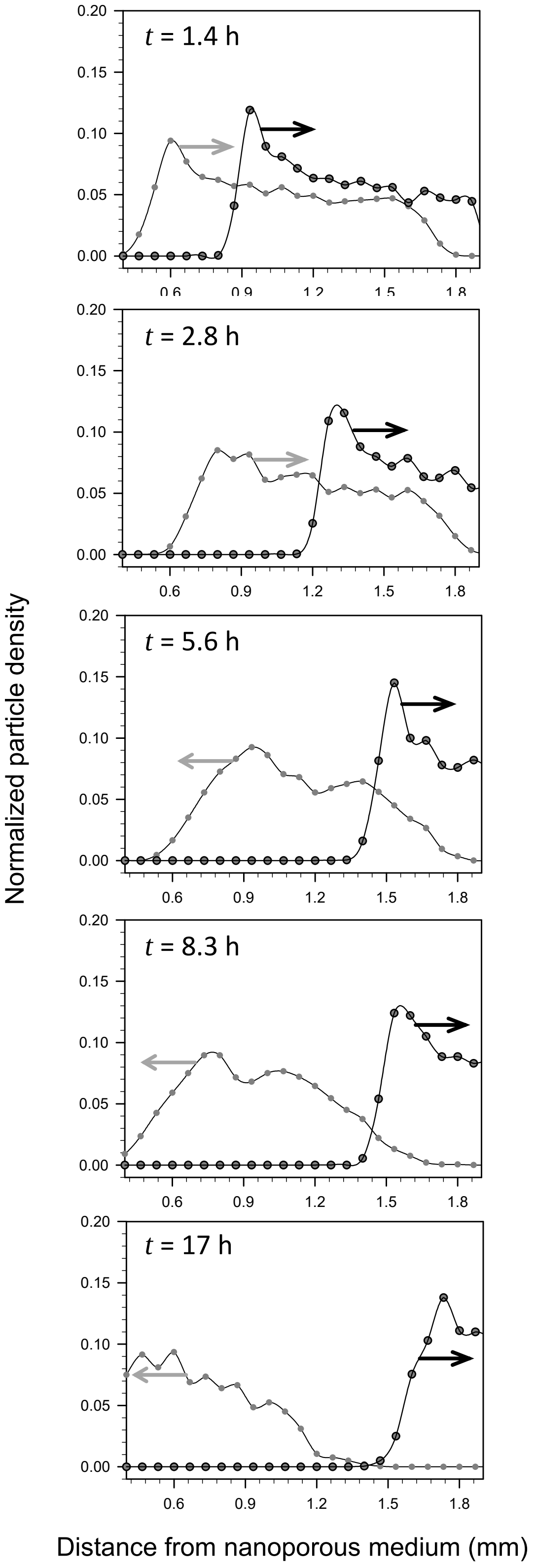

Figure S2. Histogram of two types of particles estimated from the LD simulation.

Supplementary Note 3. Numerical analysis of the time-dependent concentration profile near the nanoporous ion-exchange medium.

Although the analytic solutions in Eq. (S9-S11) well describe the concentration profiles near the nanoporous ion-exchange medium when $t < t_{lim}$, they fail to explain the recovery of the concentration gradient due to the diffusion from the bulk reservoir since the analytic solutions were based on the assumption that the bulk reservoir is located at $x = \infty$. In an actual device, however, once the concentration gradient was fully developed in the microchannel, the diffusion from the bulk reservoir flattened the concentration gradient by supplying the potassium ions. This was verified by numerical simulation for the finite distance (4.5 mm) between the ion exchange medium and the bulk reservoir. Numerical formulation was the same as the one proposed by Lee *et al.*[4]. The result showed that the concentration profiles changed their tendencies over time, which can be divided into three stages.

In the stage 1, the ion depleted region near the ion exchange medium expanded to the reservoir as shown in Fig. S3 (a). In this stage, the numerically obtained concentration profile well matched with the analytic solutions proposed by Lee *et al.* [4]. In the stage 2, protons which diffused from the surface of the medium reached the reservoir, and the protons were rapidly removed by the bulk reservoir. This caused the decrease of the total concentration and sharpened the total concentration gradient in the microchannel. In the stage 3, finally the region where potassium ions were depleted reached the reservoir and the reservoir provided the potassium ions to recover the concentration gradient of the potassium ions. This caused the increase of the total concentration and flattened the concentration gradient since the influx of potassium ions exceeded the outflux of protons ions.

Therefore, at $t > t_{diff}$, the diffusiophoretic force on a charge particle started to decrease and the particle changed its direction of motion towards the ion-exchange medium. For the

microchannel of L_2 = 4.5mm, the total concentration gradient was expected to be flat after t = 9,980 seconds. This is why one experimentally observed that the charged particle changed its direction of motion even without the help of saturated imbibition velocity as shown in Fig. S3 (b). In this case, ion exchange medium was patterned in 1-D structure but the particles changed their direction due to the decreased concentration gradient after t_{lim}.

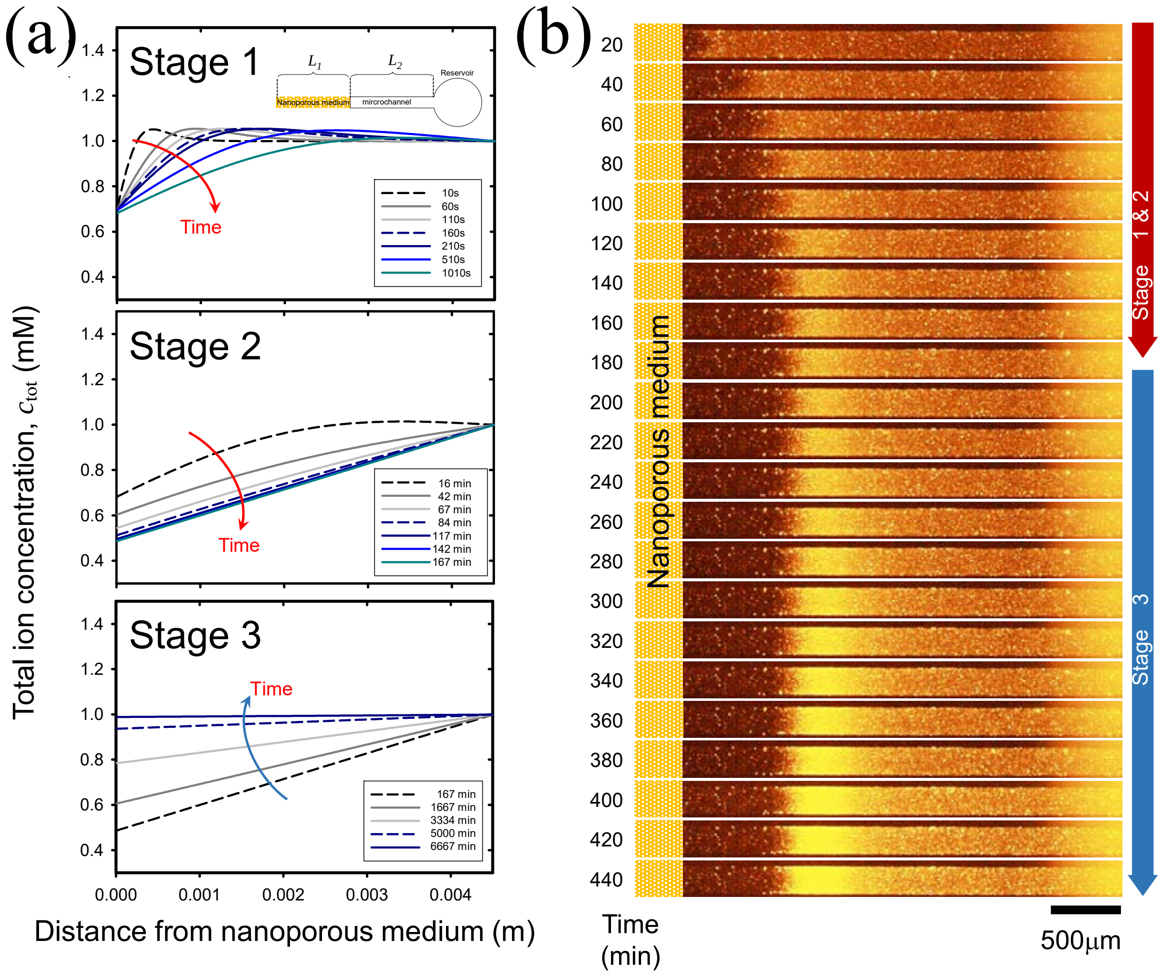

Figure S3. Recovery of the concentration profile induced by the diffusion from the reservoir. (a) Time-dependent numerical simulation on the concentration profiles in a finite microchannel, and (b) the experimental validation of the concentration recovery.

References

1 Perez-Cruz, A., Stiharu, I. & Dominguez-Gonzalez, A. Two-dimensional model of imbibition into paper-based networks using Richards' equation. *Microfluidics and Nanofluidics* **21**, doi:10.1007/s10404-017-1937-0 (2017).

2 Keh, H. J. Diffusiophoresis of charged particles and diffusioosmosis of electrolyte solutions. *Current Opinion in Colloid & Interface Science* **24**, 13-22, doi:10.1016/j.cocis.2016.05.008 (2016).

3 Lee, H., Kim, J., Yang, J., Seo, S. W. & Kim, S. J. Diffusiophoretic exclusion of colloidal particles for continuous water purification. *Lab Chip* **18**, 1713-1724, doi:10.1039/c8lc00132d (2018).

4 Lee, J. A., Lee, D., Park, S., Lee, H. & Kim, S. J. Non-negligible Water-permeance through Nanoporous Ion Exchange Medium. *Sci Rep* **8**, 12842 (2018).
